# Role of Minor Phase Morphology on Mechanical and Shape-Memory Properties of Polylactide/Bio-Polyamide Nanocomposite

**DOI:** 10.3390/polym16172413

**Published:** 2024-08-26

**Authors:** Vladislav Bondarenko, Ramin Hosseinnezhad, Andrei Voznyak

**Affiliations:** 1 Physics and Mathematics Department, Kryvyi Rih State Pedagogical University, Gagarin av. 54, 50086 Kryvyi Rih, Ukraine; 2 Centre of Molecular and Macromolecular Studies, Polish Academy of Sciences, Sienkiewicza str., 112, 90363 Lodz, Poland; 3 Department of Technological and Professional Education, Kryvyi Rih State Pedagogical University, Gagarin av. 54, 50086 Kryvyi Rih, Ukraine

**Keywords:** shape-memory polymer composites, in situ polymer fiber generation, mechanical and shape-memory properties

## Abstract

In situ-generated nanofibrillar polymer–polymer composites are excellent candidates for the production of polymer materials, with high mechanical and SME properties. Their special feature is the high degree of dispersion of the in situ-generated nanofibers and the ability to form entangled nanofiber structures with high aspect ratios through an end-to-end coalescence process, which makes it possible to effectively reinforce the polymer matrix and, in many cases, increase its ductility. The substantial interfacial area, created by the in situ formed fiber/matrix morphology, significantly strengthens the interfacial interactions, which are crucial for shape fixation and shape recovery. Using the polylactide/bio-polyamide (PLA/PA) system as an example, it is shown that in situ PA fibrillation improves the mechanical and shape-memory properties of PLA. The modulus of elasticity increases by a factor of 1.4, the elongation at break increases by a factor of 30, and the shape-strain/fixity ratio and shape recovery increase from 80.2 to 97.4% and from 15.5 to 94.0%, respectively. The morphology of the minor PA phase is crucial. The best result is achieved when a physically entangled nanofibrous network is formed.

## 1. Introduction

Shape-memory polymers (SMPs) and their composites have attracted much research attention due to their ability to assume one or more temporary shapes and to recover their original shape (or other temporary shapes) under the influence of external factors [1,2,3,4]. The recovery of the original shape can be triggered by heat, light radiation, electric current, magnetic fields, moisture, or high-frequency waves [5,6,7,8]. Despite the wide variety of effects, heat (direct or indirect) is most commonly used to program and recover shape.

Currently, a wide range of polymers can assume different shapes (thermoplastics and thermosets). The advantage of SMPs based on thermosets is their good mechanical and thermal stability beyond the glass transition temperature. The advantage of SMPs based on thermoplastics is the possibility of producing biocompatible, biodegradable materials. Thermoplastic polymers with such properties include polyurethane, polylactide, and others. However, the low mechanical strength of thermoplastic-based shape-memory materials in the elastic state prevents their widespread use in industries such as biomedicine, aerospace, and many others.

One solution to the problem was to incorporate various nanofillers into the polymer matrix, e.g., carbon nanotubes, carbon nanoplatelets, nanocellulose, and nanoclays [9,10,11,12]. However, the disadvantage of this approach was the deterioration of the mobility of the polymer chains, leading to both a decrease in the plasticity of the reinforced polymers and a reduction in shape-memory properties, such as shape-fixation and shape-recovery rate.

Another approach involves the use of in situ-generated nanofibrillar polymer–polymer composites (NFCs) [13]. This method, pioneered by Fakirov et al., is based on the understanding that stretching polymers enhances their mechanical properties [14]. These composites are formed by fibrillating the dispersed phase of an immiscible polymer blend [15,16,17,18,19,20]. The in situ-generated NFCs have several advantages over the conventional polymer–polymer composites based on ready-made polymer nanofibers. Firstly, it is the high degree of dispersion of the in situ-generated nanofibers and the associated high specific surface area that significantly improve their mechanical properties. Secondly, there is the possibility of forming entangled nanofiber structures with a high aspect ratio through an end-to-end coalescence process. From the perspective of SMP fabrication, in situ-generated NFCs have high strength and ductility, and entangled high-aspect-ratio nanofiber structures create additional net points by increasing the fraction of interfaces that contribute to the entanglements and interactions between the molecular chains.

**Figure 1:**
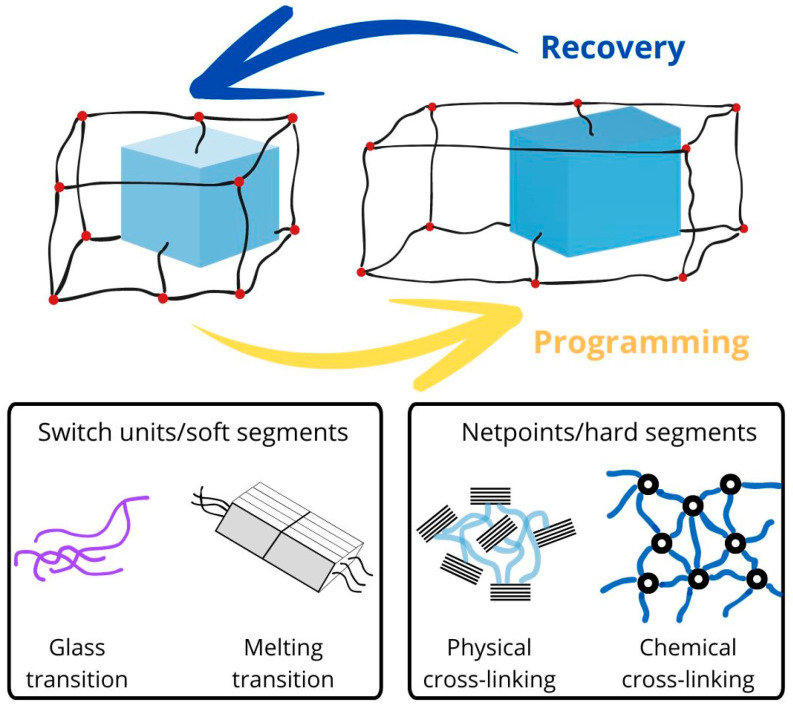
Schematic illustration of SMPs architecture (Adapted with permission from [22]. Copyright (2010) Royal Society of Chemistry).

Theoretically, SMPs have two domain types (Figure 1): switching domains, also known as “a reversible phase”, “switching units”, and “soft segments,” and permanent domains, also known as “net points”, “hard segments”, and “fixed phase” [21,22]. The permanent domains can be generated from polymer crystals, chain entanglements, and cross-links of molecular chains. The former store entropic elasticity and are therefore responsible for switching to the original or temporary shapes when the right stimulus is applied, while the latter fix the temporary shape(s) since it exhibits sensitivity to certain stimuli. The latter is not sensitive to these stimuli and therefore works to fix the temporary shape. Its task is to prevent long-range macromolecular movements. In the case of NFCs, the switching domains can therefore be in situ-generated nanofibers and the polymer matrix can serve as permanent domains, or conversely, the polymer matrix can be switching domains and in situ-generated nanofibers can serve as permanent domains.

To be best of our knowledge, there is only one paper that has investigated the shape-memory properties of in situ-generated NFCs [23]. In particular, it was shown that in situ poly(glycolic acid) fibrillation can improve the recovery properties of poly[(L-lactide)-co-(ε-caprolactone)]; in fact, the shape-recovery rate increased from 80.5 to 93.2%.

In this work, the effect of in situ bio-polyamide (PA) fibrillation on the shape-memory properties of 95:5 PLA/PA blends in terms of morphology, thermal and mechanical properties was investigated to correlate them with the shape-memory performance of the nanocomposites. The morphology of the PA minor phase changed from nanodroplets to disentangled nanofibers and then to entangled nanofibers forming a continuous network. This innovative approach offers the flexibility to adjust the number and combination of immiscible polymer components, opening up new possibilities for developing high-performance SMPs. Additionally, the ability to fine-tune the properties of these composites by adjusting the minor phase morphology could lead to the development of customizable shape-memorial materials for potential specific applications in medical devices, self-healing materials, and biodegradable packaging that requires shape retention. 

## 2. Experimental

### 2.1. Materials

A commercially available polylactide grade, PLA 3215D, was purchased from NatureWorks LLC (Dortmund, Germany) and used as the matrix. This grade of PLA is biodegradable; it has a density of 1.24 g/cm^3^ and a molecular weight of 120,000 g/mol. It has a glass transition temperature of 61 °C. A fully bio-based polyamide (PA) with the trade name Vestamid (Shanghai, China), supplied by Evonik Industries (Essen, Germany), was used to produce the composite materials. The monomers for the synthesis of this type were obtained from castor oil and have a melting point of 200 °C. The melting point of PA is 200 °C, and its equilibrium melting temperature is 238 °C. Joncryl ADR4400, a polymeric chain extender with a medium epoxy equivalent weight, was provided by BASF Corporation (Ludwigshafen, Germany) and used to improve the viscoelasticity of PLA and its interaction with PA.

### 2.2. Sample Preparation

PLA/PA blends and nanocomposites were prepared using the procedure detailed in [24]. To produce the blend PLA/PA (95/5), 5 wt.% PA was melt-blended with PLA (both components were dried for 8 h at 60 °C) and the chain extender in a twin-screw extruder, whereby the temperature zones were increasingly adjusted from 200 °C to 230 °C. The blend was also processed in a single-screw extruder with a temperature gradient from 230 °C (feed zone) to 175 °C (slit die). A co-rotating twin-screw extruder 2 × 20/40D EHP (Zamak Mercator, Skawina, Poland) operating at 120 rpm was used for processing. After fibril formation, the tapes were extruded using a single-screw extruder (PlastiCorder PLV 151, Brabender (Duisburg, Germany); D = 19.5 mm, L/D = 25 and 20 rpm), which was equipped with a 12 mm wide, 0.8 mm thick and 100 mm long slot die. The process pressure in the slitting tool was 20.1 MPa. The zone temperature was specifically set to initiate the crystallization of the deformed minor polymer phases (i.e., PA) during the shearing process. The extruder was equipped with a slit die to increase the residence time. The extrudates coming out of the single-screw extruder were poured onto a conveyor belt at room temperature without additional drawing. The extrudates were obtained in the form of tapes approximately 0.5 mm thick and 10 mm wide. 

### 2.3. Mechanical and Thermal Properties

An Instron-5582 (Universal Testing Machine, High Wycombe, UK) was employed to evaluate the tensile properties of PLA, blend, and composite at a strain rate of 5%/min as per ISO 527-2 standards [25]. Additionally, a Q800 DMA (TA Instruments, New Castle, DE, USA) was used to measure the thermomechanical characteristics of rectangular samples (24 × 10 × 0.75 mm³) at a heating rate of 2 °C/min. To study the thermal properties, a DSC Q20 differential scanning calorimeter (TA Instruments) was used to heat samples under a dry nitrogen atmosphere from 0 °C to 250 °C at a rate of 10 °C/min.

### 2.4. Scanning Electronic Microscopy (SEM)

The morphology of the blend and composite, cryogenically fractured along the extrusion direction and coated with gold, was investigated with a JEOL JSM-5500 LV (Scanning Electron Microscope, Tokyo, Japan).

### 2.5. Shape-Memory Characterization Test 

The characterization of thermally activated shape memory of samples was performed using the same Q800 DMA instrument with the film tension clamp under controlled strain and force. For this purpose, samples were heated to 60 °C and then deformed to the strain value of 10%. Once cooled down to room temperature, the strain was released. The samples were then reheated to 60 °C, and their recovery was recorded.

To quantitatively assess the thermally activated shape-memory behavior of the samples, the shape-fixity ratio (R_f_) and shape-recovery ratio (R_r_) were determined. The R_f_ ratio, which measures how well the material preserves its temporary shape, is calculated as the fraction of the strain that remains fixed relative to the total strain, as demonstrated by the following equation: *R*_*f*_ = (ε_*un*_ − ε_*max*_) × 100%. R_r_, the ability to recover the initial shape, was considered as the ratio of the recovered strain to the total strain according to the following equation: *R*_*r*_ = ((ε_*un*_ − ε_*fin*_)/ε_*max*_) × 100%, where ε_un_ is the strain after cooling and unloading, ε_max_ is the strain obtained before the constant loading was released, and ε_fin_ is the strain obtained after heating in the step of recovery.

### 2.6. In Situ 2D Small-Angle X-ray Scattering (SAXS)

The test was facilitated by employing a home-assembled in situ SAXS apparatus, where a Linkam MicroTester, TST350 (Tadwarth, UK) was connected to a GeniX Cu-LD (Xenocs SAXS Instrument, Sassenage, France). Online stress–strain curves enabled the precise stretching of dumbbell-shaped samples at a strain rate of 1% min^−1^. At specific strains, stretching was paused shortly to capture high-quality patterns with minimum inevitable stress relaxation during the acquisition. 

## 3. Results and Discussion

### 3.1. Morphology

It is known that in polymer blends in the molten state, the efficiency of the transition from droplets to fibers under shear deformation is determined by two factors: viscosity and elasticity ratios [26]. The morphology of the minor phase can therefore be changed by varying the viscosity and the elasticity ratios. The higher the viscosity and elasticity of the matrix, the greater the degree of deformation and extension of the dispersed droplets, and the lower the elasticity of the dispersed polymer, the stronger the stabilizing effect that prevents the droplets from breaking. As a result, lower viscosity and elasticity ratios favor the formation of a fibrillar morphology. In this work, a PLA chain extender blend was used to increase the viscosity and elasticity of PLA.

**Figure 2:**
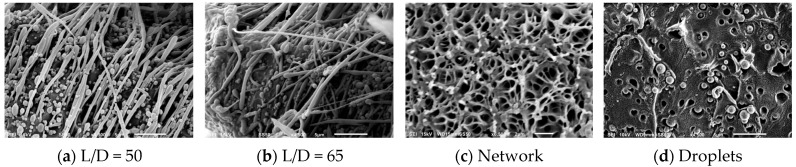
SEM images of PLA/PA in situ-generated composites. The concentration of Joncryl, respectively, is (**a**) 0, (**b**) 1, (**c**) 2 and (**d**) 4%. L/D is the characteristic ratio between the length of the fibrils and their thickness (calculated based on the visible part of the fibrils and may therefore be slightly lower than the actual values).

Figure 2 shows the effects of viscoelasticity on the fibrillar morphology of PA. The PA nanofibers became visible after a light etching of the matrix from the composites produced in situ. In a PLA/PA blend (Figure 2a) with a viscosity and elasticity ratio of 2.8 and 15, respectively, the formation of fibrils with a diameter of 300 to 900 nm was observed. Lower values of the viscosity and elasticity ratio, 0.4 and 3, respectively, in the case of PLA/PA/1%J, led to the formation of thinner fibrils with diameters in the range of 200–700 nm (Figure 2b). At the same time, the nanofibers tended to be more uniform. A further reduction of the viscosity ratio to 0.3 and the elasticity ratio to 2 for PLA/PA/2%J was accompanied by an even greater thinning of the fibrils, which varied between 100 and 300 nm. It should be noted that in this case, a physically entangled nanofibrous network started to form (Figure 2c). The latter could be because thinner and longer nanofibrils increase the probability of forming physical connections between them. However, as the viscosity ratio approaches 0.15 with a constant elasticity ratio of about 2 for PLA/PA/4%J, the nanofibers begin to break up into tiny nanodroplets. Figure 2d shows that the newly formed subparticles have a diameter of 100 to 500 nm. Apparently, there is a certain critical thickness of the nanofibrils below which the flow of the molten PA inclusions becomes unstable due to the excessively high stress at the interface.

### 3.2. Mechanical Properties

**Figure 3:**
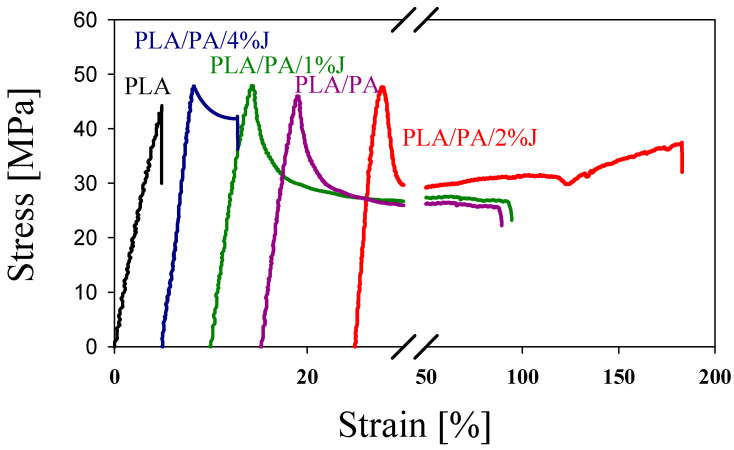
Stress–strain dependencies of neat PLA and an in situ-generated composites of PLA/PA. The curves are shifted horizontally for clarity.

The transformation of the morphology of the minor phase leads to a significant change in the mechanical properties of PLA/PA blends. Figure 3 shows the stress–strain dependencies determined in the uniaxial tensile test for the PLA and PLA/PA in situ-generated composite. It can be seen that the formation of thinner and longer nanofibers (increased L/D ratio) leads to an increase in the modulus of elasticity and tensile strength as well as an increase in elongation at break. The best result is achieved when the nanofibers form a physically entangled network (Table 1). However, a further reduction in the diameter of the nanofibers, which leads to a loss of their stability and a transition to a drop-shaped morphology, has a negative effect, as this reduces both the strength and the ductility of the PLA/PA composite.

**Figure 4:**
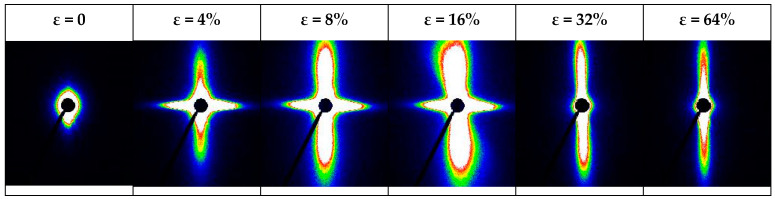
2D-SAXS patterns captured at specific strains during the uniaxial tensile deformation of PLA/PA/2%J nanocomposite at room temperature. Warmer colors (like red) signify higher scattering intensity, while cooler colors (like blue) represent lower intensity.

The SAXS technique was also employed to explore the deformation mechanism of the PLA/PA/2%J nanocomposite, which was considered to differ from the microcracking known for neat PLA. The 2D-SAXS patterns of the PLA/PA/2%J nanocomposite were captured at specific strains during the uniaxial tensile deformation and at room temperature. The pattern of the initial undeformed nanocomposite in Figure 4 is uniform in all orientations and rules out any possibility of oriented structures. Once the specimen is stretched to the strain of 4%, before the yielding point, two intense perpendicular strips emerge in the pattern.

**Figure 5:**
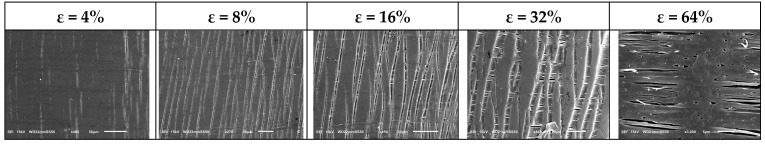
SEM micrographs of PLA/PA/2%J nanocomposite upon tensile test, revealing intensive heterogeneous tensile and shear crazing, as well as the orientation of voids.

The strips, independent of volume changes, remain normal compared to each other, despite further stretching the sample where it undergoes yielding (ε = 8%) and plastic deformation (ε = 16%). This reflects the character of simultaneous tensile and shear crazing, contributing to the inhomogeneous plastic deformation of PLA-based nanocomposites, shown in Figure 5. A gentle increase in the intensity of streaks by increasing the strain from 4% to 16% hints at an escalation in the nucleation density of crazes due to the sites, resulting from dormant shear crazes—more information can be found elsewhere [26]. However, the evolution of the crazes in the localized shear bands is retarded due to the spanned edges of crazes by the network of nanofibers. At higher strains, before strain hardening, the orthogonal strips transform into a stable vertical pattern, which demonstrates the orientation of voids in shear bands along the extension direction.

### 3.3. Thermal and Dynamic Mechanical Analysis

**Figure 6:**
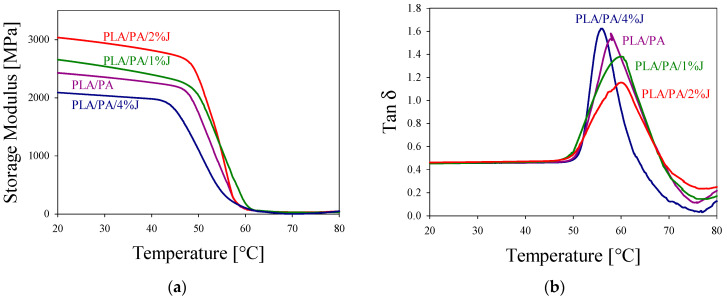
(**a**) Storage modulus and (**b**) tanδ of PLA/PA in situ-generated composites as functions of temperature.

Figure 6a presents the DMA curves for the PLA/PA composites. It is evident that the formation of thinner and longer PA fibers results in an increased storage modulus (E’) in the region below T_g, PLA_ (see Figure 6a).

To explore phase interactions, tan δ curves were examined. Figure 6b reveals that the composites exhibit a tan δ peak within the temperature range of 50–80 °C, with variations in tan δ values indicating changes in internal friction corresponding to the PA phase morphology. This behavior is likely due to physical cross-linking between PLA and PA molecular chains at the phase interfaces. The PA phase, when structured as nanofibrils rather than nanodroplets, creates a network with more physical entanglements with PLA, which more effectively restricts the viscous flow of PLA molecular chains.

To assess the extent of interaction between the fibers and the matrix, the following expression can be applied [27]:

(tan δ_max_)_c_ = (tan δ_max_)_m_ − αV_f_

where (tan δ_max_)_c_ and (tan δ_max_)_m_ are the peak values of tanδ of the in situ-generated composites and the matrix, respectively; V_f_ is the volume fraction of the filler; and α is a coefficient. According to [27], the greater the value of α, the stronger the interaction between the fillers and the matrix. The values of α for the different PLA/PA composites considered in this study were calculated and are listed in Table 2. As presented, the value of α is highest for PLA/PA/2%J.

Comparative analysis of the DMA curves reveals that composites with PA phases organized as nanofiber networks form more effective physical cross-linking at the interfaces. This results in superior shape-memory effect (SME) parameters, including enhanced shape-recovery and shape-fixation ratios. Other research has also emphasized the critical role of physical cross-linking at phase interfaces in promoting shape recovery [28,29,30].

**Figure 7:**
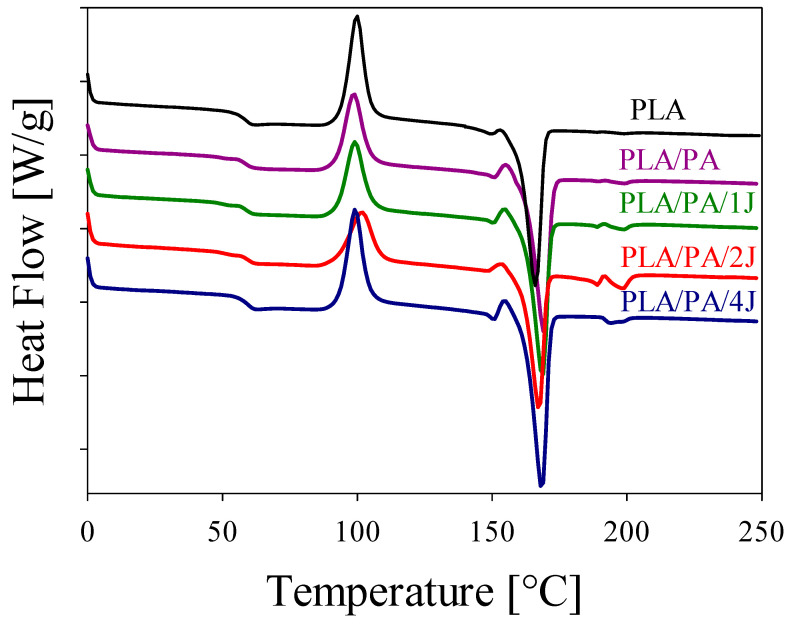
Melting endotherms of PLA/PA in situ-generated composites.

Furthermore, the quantity of crystalline PA phases, which function as permanent domains for shape-memory effect (SME) programming, is crucial. Figure 7 illustrates the DSC curves of the PLA/PA in situ-generated composites after the heating cycle. It can be seen that the change in PA morphology influences the degree of crystallinity of PLA, χ_c_. Composites containing a network of PA nanofibers exhibit the highest degree of crystallinity (Table 2). It is worth also highlighting the fact that the T_g_ value depends on the interaction between PLA and PA in the blends, which is limited and results in minimal changes in the thermal transition properties. However, the presence of the compatibilizer in the PLA/PA/2%J blend with improved morphology and interaction promotes better interfacial adhesion between the PLA and PA phases. This enhanced interaction leads to a greater effect on the glass transition, thereby altering the T_g_ in this specific blend.

It should be noted that any subtle discrepancy between the T_g_ results from DSC and DMA can be attributed to the different operating conditions and sensitivities of these techniques. The DSC measurements were conducted at a heating/cooling rate of 10 °C/min, which provides an overview of the bulk thermal properties, including the glass transition. However, this relatively fast rate may average out subtle transitions, leading to T_g_ values that appear less affected by changes in the microstructure. In contrast, DMA was performed at a slower rate of 2 °C/min, which allows for a more precise detection of changes in the material’s viscoelastic properties. This slower rate enhances the sensitivity of the DMA to mechanical responses, revealing shifts in T_g_ that reflect alterations in the microstructure, such as fibrillar network morphology within the matrix. 

### 3.4. Shape-Memory Properties

**Figure 8:**
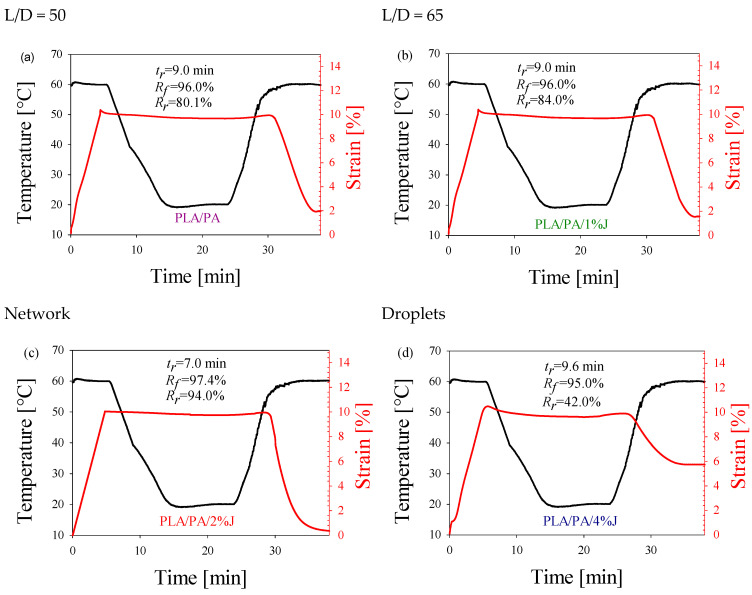
Temperature and strain profiles of PLA/PA composites upon the shape-memory cycle. The concentration of Joncryl in the composites with the minor phase’s morphology of (**a**) fibrillar (L/D = 50), (**b**) fibrillar (L/D = 65), (**c**) network and (**d**) droplet was 0, 1, 2 and 4%, respectively. The deformation temperature (T_d_) was 60 °C.

For the shape-memory effect at the temperature T_g, PLA_ was programmed to determine the characteristics of the processes for fixing the temporary and recovering the original shape for the PLA/PA in situ-generated composites (Figure 8). It can be seen that the morphology of the minor PA phase affects the shape-memory properties of composites. The highest values of the strain-fixity ratio R_f_ and the strain-recovery ratio R_r_ are observed for PLA/PA in situ-generated composites with an entangled nanofibrous network morphology of the minor PA phase (Table 3). The low shape-recovery ratio of pure PLA could be due to two factors: (a) the difference between the deformation (programming) temperature and the glass transition temperature and (b) the amount of strain applied. The further the deformation temperature is from the glass transition temperature, the lower the recovery ratio. Since the deformation temperature in our study was adjusted to the glass transition temperature of the composites, this was not the optimal temperature for neat PLA.

The physical entanglement network at the PLA–PA interfaces tends to be weak for the droplet matrix morphology. This weakness allows PLA chains to slip during the temporary shape formation, impairing their ability to fully recover to the original shape upon reheating. Conversely, longer and thinner PA nanofibers form a more robust network of physical entanglements, creating stable permanent domains. This enhanced network significantly improves both shape-fixation and -recovery rates. In addition, the increase in the number of permanent domains is also associated with an increase in the number of PLA crystals (an increase in the degree of crystallinity) during the formation of the PA nanofibers. The shape-recovery time is lowest for composites containing entangled nanofiber networks.

## 4. Conclusions

In this work, the SME and mechanical properties of in situ-generated polymer–polymer composites are investigated using the example of the PLA/PA system. It has been shown that such systems can exhibit a good combination of high strength, plasticity and SME properties. The morphology of the dispersed phase is pivotal to achieving optimal performance. The best mechanical and shape-memory effect (SME) properties are attained when an in situ network of entangled nanofibers is generated. The result obtained is related to the formation of a larger specific contact area between the polymer phases, which contributes to the formation of a better complex of mechanical properties and better shape-memory performance. In particular, the in situ-generated PLA/PA composite exhibits a 1.4-fold higher modulus of elasticity and a 1.2-fold higher yield strength as well as a 30-fold higher elongation at break compared to the original PLA. The shape-fixity ratio R_f_ and the shape-recovery ratio R_r_ increase from 80.2 and 15.5 for PLA to 97.4 and 94.0% for the in situ-generated PLA/PA composite. The shape-recovery time is reduced from 9.7 to 7.0 min.

## Figures and Tables

**Table 1:** Mechanical properties of in situ-generated composites of PLA/PA.

Composite	Modulus of Elasticity, GPa	Yield Stress, MPa	Stress at Break, MPa	Strain at Break, %
PLA	1.50	42.1	30.0	5
PLA/PA	1.90	43.4	25.8	70
PLA/PA/1%J	1.98	49.2	27.9	80
PLA/PA/2%J	2.12	49.5	39.8	150
PLA/PA/4%J	1.72	48.2	43.3	13

**Table 2:** Values of α and χ_c_ for some composites.

Composite	α	χ_c_, Degree of Crystallinity, %
PLA/PA	3.63	15.0
PLA/PA/1%J	5.34	15.9
PLA/PA/2%J	8.13	19.2
PLA/PA/4%J	2.12	12.8

**Table 3:** Shape-memory properties of in situ-generated composites of PLA/PA.

Material	Shape-Fixity Ratio R_f_, %	Shape-Recovery Ratio R_r_, %	Shape-Recovery Time, min
PLA	80.2	15.5	9.7
PLA/PA	96.0	80.1	9.0
PLA/PA/1%J	96.0	84.0	9.0
PLA/PA/2%J	97.4	94.0	7.0
PLA/PA/4%J	95.0	42.0	9.6

## Data Availability

The original contributions presented in the study are included in the article, further inquiries can be directed to the corresponding author.
